# Astrochemistry and Astrobiology: Materials Science in Wonderland?

**DOI:** 10.3390/ijms20174079

**Published:** 2019-08-21

**Authors:** Marco d’Ischia, Paola Manini, Marco Moracci, Raffaele Saladino, Vincent Ball, Helmut Thissen, Richard A. Evans, Cristina Puzzarini, Vincenzo Barone

**Affiliations:** 1 Department of Chemical Sciences, University of Naples “Federico II”, Complesso Universitario di Monte S. Angelo, Via Cupa Nuova Cinthia 21, 80126 Naples, Italy; 2 Department of Biology, University of Naples “Federico II”, Complesso Universitario di Monte S. Angelo, Via Cupa Nuova Cinthia 21, 80126 Naples, Italy; 3 Institute of Biosciences and BioResources, National Research Council of Italy, Via P. Castellino 111, 80131 Naples, Italy; 4 Department of Ecological and Biological Sciences, Via S. Camillo de Lellis, University of Tuscia, 01100 Viterbo, Italy; 5 Institut National de la Santé et de la RechercheMédicale, 11 rue Humann, 67085 Strasbourg Cedex, France; 6 Faculté de Chirurgie Dentaire, Université de Strasbourg, 1 Place de l’Hôpital, 67000 Strasbourg, France; 7 Commonwealth Scientific and Industrial Research Organisation (CSIRO) Manufacturing, Clayton, VIC 3168, Australia; 8 Department of Chemistry “Giacomo Ciamician”, University of Bologna, Via F. Selmi 2, I-40126 Bologna, Italy; 9 Scuola Normale Superiore, Piazza dei Cavalieri 7, I-56126 Pisa, Italy

**Keywords:** prebiotic processes, polycyclic aromatic hydrocarbons, aminomalononitrile, formamide, volcanic fumaroles, computational methods, solid-state photochemistry, surface functionalization, nanostructures, hybrid functional materials

## Abstract

Astrochemistry and astrobiology, the fascinating disciplines that strive to unravel the origin of life, have opened unprecedented and unpredicted vistas into exotic compounds as well as extreme or complex reaction conditions of potential relevance for a broad variety of applications. Representative, and so far little explored sources of inspiration include complex organic systems, such as polycyclic aromatic hydrocarbons (PAHs) and their derivatives; hydrogen cyanide (HCN) and formamide (HCONH_2_) oligomers and polymers, like aminomalononitrile (AMN)-derived species; and exotic processes, such as solid-state photoreactions on mineral surfaces, phosphorylation by minerals, cold ice irradiation and proton bombardment, and thermal transformations in fumaroles. In addition, meteorites and minerals like forsterite, which dominate dust chemistry in the interstellar medium, may open new avenues for the discovery of innovative catalytic processes and unconventional methodologies. The aim of this review was to offer concise and inspiring, rather than comprehensive, examples of astrochemistry-related materials and systems that may be of relevance in areas such as surface functionalization, nanostructures, and hybrid material design, and for innovative technological solutions. The potential of computational methods to predict new properties from spectroscopic data and to assess plausible reaction pathways on both kinetic and thermodynamic grounds has also been highlighted.

## 1. Introduction

Until not too long ago, the terms astrochemistry and astrobiology conveyed a general impression of niche research fields at the boundaries of science, relying heavily on theoretical analysis and speculation rather than solid experimental evidence. This negative attitude and the underlying skepticism or prejudice toward the chemistry of astrophysical environments was due in part to the limited credit given to theories based on spectroscopic analysis of emissions from remote regions of space, and in part to the widely held belief that space is not a place where useful and interesting chemical processes may occur in a substantial, verifiable, and repeatable manner. Space was traditionally perceived as a hostile and barren system with adverse conditions (exceedingly low temperature, very low pressures, highly energetic or ionizing radiation) hindering the development of a consistent molecular chemistry beyond the di-or tri-atomic level.

In recent decades, this negative attitude has been gradually reversed. Today, astrochemistry and astrobiology are no longer the realm of “forbidden” or “exotic” chemistry and biology, but they are increasingly being appreciated as an unexplored source of inspiration for new chemical processes, new reaction conditions, and even new concepts and theories for the study of extreme living systems. The chemistry of carbon is expected to follow the same general rules in the cosmos. Organic compounds permeate virtually all astrophysical environments as they take part in a complex cycle commencing with the outflow of matter from dying stars, through the diffuse interstellar medium and dense molecular clouds where stars and planetary systems form. Tracking the origin of organic compounds in interstellar, planetary, and prebiotic contexts is a challenge of increasing momentum that may push chemistry and, more specifically, organic chemistry towards new scientific boundaries and technological opportunities. 

Researchers now routinely look to biology for inspiration in regard to concepts and chemistry that can be exploited in materials science. The aim of this paper was to pick up a few representative examples of chemical processes of astrochemical and prebiotic relevance, and to discuss them as a source of inspiration for new materials or molecular systems, or for little explored chemical reactions and methodologies deserving of further research in materials science and technology.

## 2. Complex Organic Molecules

### 2.1. Polycyclic Aromatic Hydrocarbons (PAHs)

PAHs are a broad class of aromatic hydrocarbons made up of fused benzene rings. Interest in the astrochemistry of PAHs derives from their detection in various astronomical environments, including cosmic dust, icy satellites, carbonaceous meteorites, comets, and so on. PAHs have also been implicated in putative prebiotic processes involved in abiogenesis and embraced within the so-called “PAH world hypothesis”.

Though this hypothesis has received much criticism, it has the merit of drawing attention to the potential relevance of PAHs in the generation of organic components in astrochemical environments that may be precursors to a broad range of species, including simple biomolecules.

The evidence in support of the occurrence of PAHs in the astronomical environments is summarized in what is commonly referred to as “the PAH hypothesis”, which was originally proposed by Puget andLéger in 1984 [1] and Allamandola et al. in 1985 [2]. The hypothesis states that the infrared emissions from many astronomical objects are compatible with those of PAHs. Support for the PAH hypothesis came mainly from: (a) the banded rather than continuous nature of the emission spectra from various sources; (b) the close association of the emissions with ultraviolet radiation, suggesting that they are due to gas phase molecules excited by photons rather than thermal emissions from solid materials [3,4]; (c) the correlation between the fraction of the total infrared energy that is emitted from planetary nebulae through these features and the amount of available carbon; and (d) the correlation between the features of variating emission bands, which is in accord with a single class of chemical species being responsible for these spectral signatures. However, this hypothesis has been questioned over the years based on a number of issues, including a poor match between the experimental and/or theoretical spectra and the astronomical spectra, and a significant variability in band profiles from interstellar *vs* circumstellar emissions. Moreover, and most critically, pure PAHs alone cannot account for certain specific spectral data, e.g., the position of the 6.2 μm band, which is not compatible with the strongest C–C stretching of pure PAHs.

Several attempts have been made to improve the matching of astronomical infrared (IR) emissions with model systems. These include:

(a) hetero-atom substitution [5,6];
(b) aggregation in clusters [7,8];
(c) random mixtures of PAHs and their cations [9];
(d) delocalized π domains confined in disordered carbon mixed-phase aggregates [10].

Whatever the actual nature of the IR-emitting species, most likely a combination of different entities rather than a homogeneous class of molecules, it is of considerable relevance to materials science to investigate the possible origins of PAHs in astrophysical environments. Both bottom-up and top-down approaches have been proposed, which have highlighted an extraordinarily rich chemistry under low temperature conditions. Representative examples of the former include: (a) the vinylacetylene pathway to naphthalene via attack of ethynyl radical to ethane in the interstellar medium (ISM) [11], and (b) the etching of graphene on the surface of SiC dust grains in ISM [12]. The latter hypothesis envisions the formation of complex aromatic species on the graphitized surface of silicon carbide upon exposure to atomic hydrogen in ultra-high vacuum chambers under pressure and temperature conditions mimicking those of the ISM.

Other clues to new materials are related to the formation and modification of oxygenated derivatives of PAHs, herein referred to as oxyPAHs, which may be produced in cosmic ice analogs even at temperatures as low as 15 K degrees.

**Figure 1:**
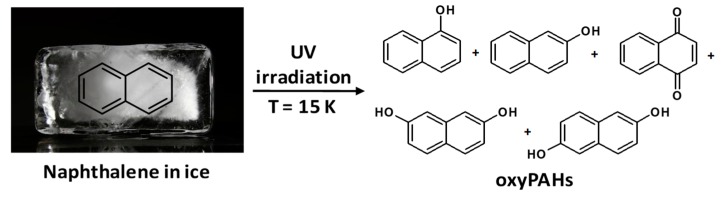
Main products identified from the UV photolysis of an H_2_O: naphthalene ice at 15 K.

OxyPAHs recently captured the interest of scientific community for their water–ice matrix photochemical reactivity in water–ice matrix in the ISM. Furthermore, oxyPAHs are interesting molecules in the study of the origin of life for their prebiotic potential. However, their stability and transformation pathways under astrophysical relevant conditions have remained largely unexplored. Extensive literature data indicate that PAHs and oxyPAHs undergo photolysis under low temperature conditions with partial structural degradation (erosion) and loss of CO_2_ [13,14]. Allamandola et al. (2001) identified 1,4-naphthoquinone in addition to 1-naphthol among the products of photolysis of naphthalene in water–ice (Figure 1) [14,15].

This observation suggests that oxygenated and oxidized polymers of PAHs may be components of the organic matter in dust grains and refractory mantles. Furthermore, transformations based mainly on oxidative polymerization may also occur to give novel species of possible relevance to so-called complex organic matter. Particular interest in this connection is related to the photochemical and autoxidation behavior of oxyPAHs, e.g., hydroxynaphthalenes following adsorption on forsterite or anatase, which gives rise to hybrid inorganic–polymer systems (see Section 3.1, below).

**Figure 2:**
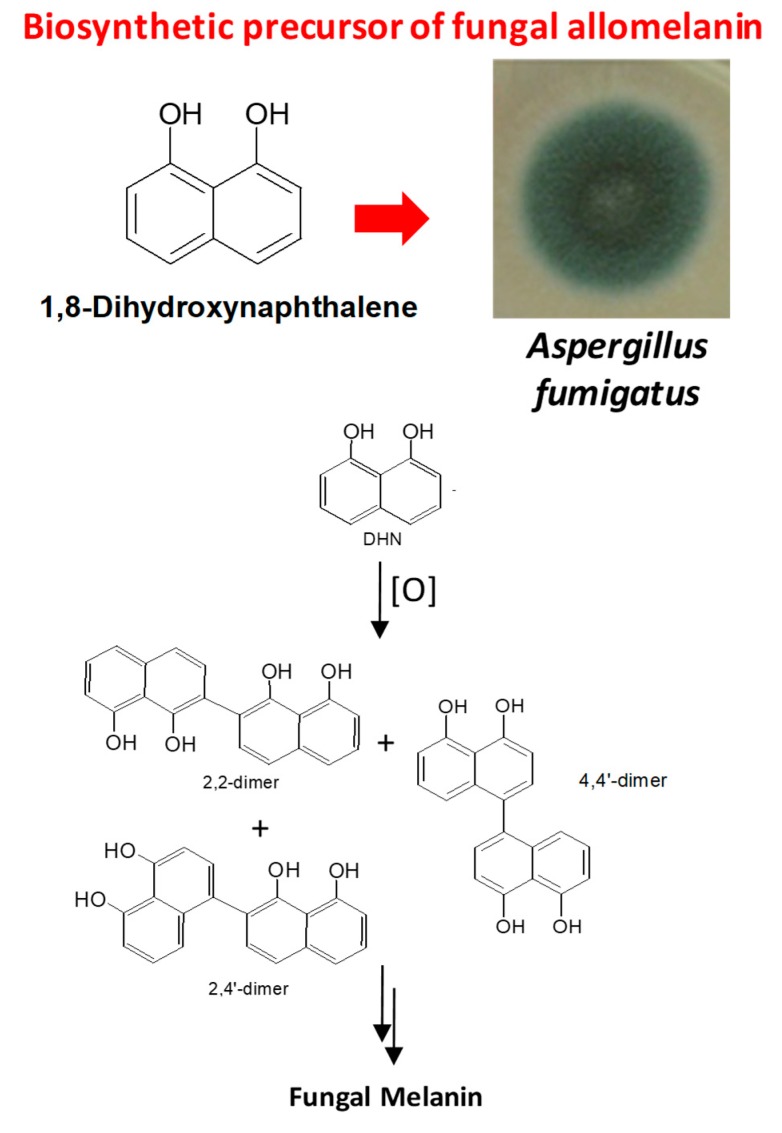
1,8-Dihydroxynaphthalene as precursor of allomelanins in fungi.

Recent studies have indicated that a representative oxyPAH, 1,8-dihdroxynaphthalene (1,8-DHN), undergoes polymerization to give a black, solid polymer mimicking fungal melanin (Figure 2) and displaying considerable H-atom donor behavior, coupled with strong chemical robustness toward hydrogen peroxide degradation and bleaching [16,17,18]. Thus, oxidative polymerization of hydroxylated PAHs may lead to allomelanin-like materials with suitable properties for biointerfaces, organic electronics, and theranostics [19]. Suitable methodologies for polymerization may be derived from solid-state oxidation, e.g., under ammonia-induced conditions [20], or ice photochemistry [15]. Under both conditions, a higher degree of structural regularity would be anticipated compared to aqueous solution chemistry, which may provide a useful means of controlling and tailoring physicochemical properties for specific applications.

### 2.2. Hydrogen Cyanide (HCN) Oligomers and Polymers: Aminomalononitrile

Polymeric derivatives of HCN may have been among the earliest macromolecules on Earth [21]. HCN polymers are heterogeneous solids that could be major components of the dark matter observed on asteroids, moons, planets, and, especially, comets. They are basically of two types: ladder structures and polyamidines, which can be hydrolyzed to polypeptides. Cleavage products of HCN polymers include amino acids, nitrogen heterocycles (purines and pyrimidines), and peptide systems [22].

The experiments performed by Urey and Miller, which aimed to mimic prebiotic chemical processes in the presence of electrical discharges [23], disclosed a complex chemical scenario relating to the ability of hydrogen cyanide (HCN), produced by an energy supply from a methane–ammonia–hydrogen- and water-containing atmosphere, to polymerize to form dimers (aminocyanocarbene), trimers (aminomalononitrile, AMN) [24], tetramers (diaminomalononitrile), and many other oligomers [25].

When solid substrates are immersed in AMN solutions in a basic medium (typically pH = 8.5), they become coated with a brown black film. The deposition kinetics of such a coating seem to be substrate-independent with an N/C atomic ratio close to 0.6 [26]. The deposition speed and the final film thickness increase when the deposition is performed in the presence of 3,4-dihydroxybenzaldehyde or 3,4,5-trihydroxybenzaldehyde [27]. All these AMN-based coatings are highly biocompatible. 

The AMN-based films, of which the structure is still unknown, present some interesting analogies with polydopamine-based coatings, obtained through the oxidation of dopamine [28] or other catecholamines like norepinephrine [29] or adrenaline [30], due to the facts that:

(i) they constitute a versatile coating technology, able to coat almost all known materials, even teflon, with a conformal nano-coating with a controllable thickness;
(ii) they have the ability to reduce metal cations like Ag^+^ [26];
(iii) they can be deposited on conductive substrates by means of cyclic voltammetry (CV)or chronoamperometry from solutions in which no chemical transformation of the monomers occur, namely at pH = 6 [31].

**Figure 3:**
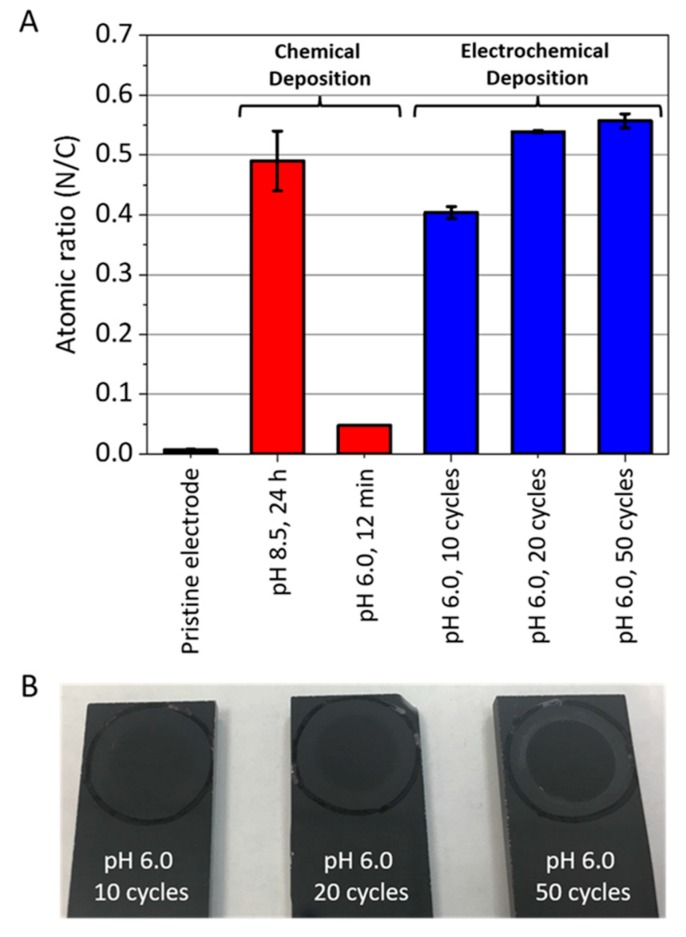
(**A**) Average nitrogen-to-carbon (N/C) elemental ratios acquired using X-ray photoelectron spectroscopy (XPS)on a pristine carbon electrode before and after chemical or electrochemical deposition of aminomalononitrile (AMN)-based films (*n* = 3, error bars represent standard deviation). All coatings were deposited in the presence of 10 mg × mL^−1^ AMN. Electrochemical deposition was performed with cyclic voltammetry (CV) at a scan rate of 50 mV × s^−1^. (**B**)Pictures of the coatings obtained on carbon electrodes after different number of CV scans performed at pH = 6.0 and at a potential sweep rate of 20 mV × s^−1^. Reproduced from Reference [31] with authorization.

The composition of the coatings obtained by electrodeposition from AMN-containing solutions at pH = 6.0 are very close to the compositions of the coatings obtained from solution at pH = 8.5 (Figure 3), as inferred using X-ray photoelectron spectroscopy (XPS).

Interestingly, the C/N ratio of the AMN-based films fell between 0.5 and 0.6 (Figure 3), significantly lower than the corresponding ration of AMN itself, namely 1. This implies the loss of nitrogen during the film formation, an important point in the determination of the reaction mechanism leading to AMN-based films.

### 2.3. Formamide-Based Prebiotic Chemistry in Phosphorylation Processes

The chemistry of HCN is strictly connected to that of formamide (NH_2_COH), the two compounds being formally equilibrated by a couple of hydration–dehydration processes. Saitta et al. in 2014 [32] described the formation of NH_2_COH from a simple gas mixture as the key intermediate in the Urey and Miller experiment, and several studies have highlighted the ubiquitarian distribution of this compound in the cosmos [33]. A robust chemical frame for NH_2_COH in the “one-pot” multi-component synthesis of biologically relevant molecules has also been reported [34]. The efficacy and selectivity of NH_2_COH chemistry in prebiotic scenarios is controlled by the chemical and physical properties of the reaction medium (e.g., mineralogical composition and morphology of the mineral environment) [35], the nature of the energy source (thermal energy, proton and heavy atom beams, photons, redox processes, etc.) [36], and the physical state of the system (gas phase, solid phase, and liquid phase supported processes) [37]. Among the prebiotic transformations, phosphorylation received particular attention, the phosphorus ester bond being involved in the backbone motif of nucleic acids, in fundamental energy pathways, and in the compartmentalization and regulatory machinery of the cell. At the origin, phosphorylation occurred on the pristine Earth (or elsewhere in the cosmos) under relatively simple conditions, using highly available (even if lowly reactive) phosphorus containing minerals as simple phosphate sources [38]. Abiotic phosphorylation processes have been reviewed, including thermal scenarios based on orthophosphate salts [39], condensed phosphates [40], and activated phosphorous intermediates produced by in situ spontaneous redox reactions [41]. Due to the unfavorable thermodynamic energy balance, these reactions have often required hard experimental conditions, as well as the presence of condensing agents and highly reactive dehydrating reagents, few of which appear to be of real prebiotic relevance [42].

Might the prebiotic chemistry of NH_2_COH open new pathways for the development of sustainable and environmentally friendly phosphorylation procedures? Pioneering studies on thermal phosphorylation of nucleosides with sodium orthophosphate and NH_2_COH have been reported by Schofstall [43]. The extent of phosphorylation exceeded 50% of yield under the most favorable conditions after 15 days at 70 °C, to afford a panel of nucleotide isomers bearing the phosphate group bonded to both primary and secondary alcohol moieties present in the sugar scaffold [44]. Unfortunately, sodium orthophosphate is not considered to have been an available source of phosphorus on the primitive Earth, since it is expected to precipitate from aqueous environments as an insoluble salt [45].

As an alternative, the phosphorylation of nucleosides has been accomplished at low temperatures (40–60 °C) using phosphorus-containing minerals as phosphate donors in the presence of NH_2_COH [46]. The procedure encompasses the use of minerals compatible with pristine prebiotic scenarios, such as Libethenite Cu_2_(PO_4_)(OH), Ludjibaite Cu_5_(PO_4_)_2_(OH)_4_, Reichenbachite Cu_5_(PO_4_)_2_(OH)_4_, CornetiteCu_3_(PO_4_)(OH)_3_,andHydroxylapatiteCa_5_(PO_4_)_3_OH. The same minerals are widely dispersed on the surface of Earth today.

**Figure 4:**
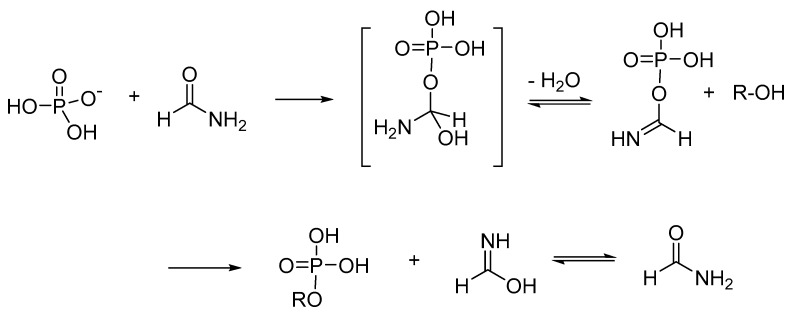
NH_2_COH-based organo-catalysis mechanism for the phosphorylation of alcohols under thermal conditions.

Spectroscopic analysis of these minerals, once pretreated with NH_2_COH at higher temperatures (130 °C, 72 h), highlighted the solubilizing effect played byNH_2_COH in the release of active free phosphate from the surface of minerals, thanks to the high value of its dielectric constant. The solubilizing effect overcomes the kinetic barrier for the approach of the substrate to the mineral surface, solving the problem of phosphate precipitation as an insoluble salt. Moreover, phosphorus-containing minerals protected the newly formed phosphorus ester derivatives from degradation. The high efficacy of the mineral phosphorylation process in the presence of NH_2_COH is a consequence of an organo-catalysis process (Figure 4).

Briefly, once delivered from the mineral to the bulk of the solution, the phosphate can react with NH_2_COH by nucleophilic acylic substitution to yield a reactive phosphorus–imide intermediate. This intermediate can successively transfer the phosphate group to the alcohol moiety, followed by the release of formamide as a formamidinic acid tautomer, which is further available for a new reaction.

Urea and choline showed a similar reactivity in the activation of soluble sodium orthophosphate, suggesting the generality of the process [47]. Thus, the formamide-based, low-temperature phosphorylation of alcohols using phosphorus-containing minerals as safe and inexpensive phosphate donor reagents might open a new path for sustainable and environmentally friendly synthesis of flame retardants and other phosphorylated polymers.

### 2.4. Biochemistry of Extremophilic Microorganisms

The discovery of extremophiles, organisms thriving only at conditions inhospitable for human beings (extremes of temperature, pH, salts, pressure, or a combination of these), and their deep rooting in the 16S phylogenetic tree of life substantially modified our understanding of the physical limits of life and opened new perspectives as to how life originated on our planet [48]. Hydrothermal vents, which host Archaea, the third domain of life after Bacteria and Eukarya, are characterized by temperatures close to the boiling point of water and pH values < 3.0, environmental conditions that are quite close to those found on Earth when life began [49]. From an astrobiological perspective, this hypothesis justifies the study of terrestrial hyperthermophilic microorganisms, and, in particular, the study of the molecular determinants of the extreme stability of the biomolecules of hyperthermophilic Archaea. In these microorganisms, membrane lipids are stabilized by ether, rather than ester bonds [50], the melting of DNA is prevented by unique nucleosides and small binding proteins [51,52], and proteins and enzymes are stabilized by compatible solutes and an increased number of ionic networks and hydrophobic interactions [53,54].

The discovery of enzymes from Archaea, showing a remarkable thermal stability coupled with resistance to extremes of pH and high concentrations of detergents, opened a new branch in biotransformation study, allowing the use of these biomolecules that showed the exquisite stereo- and regioselectivity of biocatalysts but the stability of inorganic catalysts [55]. 

## 3. Astrochemically Relevant Processes

### 3.1. Solid-State Reactions on Minerals and Meteorite Surface

Chemical reactions in the solid state display characteristic features that make them quite different from their solution or gas phase counterparts. One major advantage of solid-state organic reactions is a high degree of regio- or stereo-selectivity determined by the solid/crystal structure of the reacting species, entailing strong structural control, e.g., by crystalline lattice [56]. A number of variants can be enlisted under the broad classification of solid-state reactions, depending on the nature of the reacting system: molecular crystals, inclusion compounds in host crystals, and organic–inorganic hybrids, including thin films and coatings on mineral substrates. Being essentially out of the scope of this review, mention is given here only to topochemical reactions, which have so far been exploited for polymerization reactions. These include stepwise [2 + 2] photopolymerization of 2,5-styrylpyridine and related diolefins [57,58], thermal or radiation polymerization of diacetylenic derivatives [59,60], and the polymerization of conjugated 1,3-diene monomers, yielding highly stereoregular polymers in the form of polymer crystals [61,62].

The relevant astrochemical models for the purposes of this paper include the photochemistry of organic substrates in water–ice, mimicking dust chemistry in the ISM [13,14,15] and on mineral surfaces under a suitable atmosphere, to reproduce planetary conditions such as those on Mars. 

**Figure 5:**
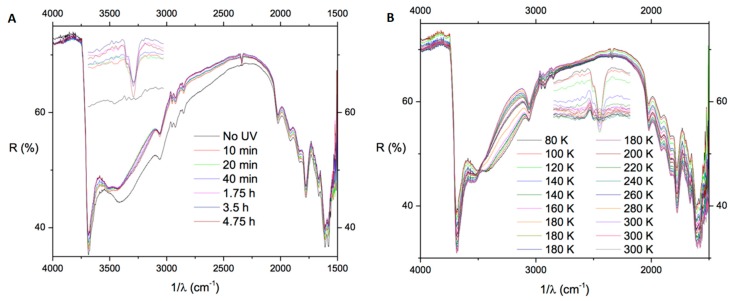
Diffuse reflectance infrared Fourier transform spectroscopy (DRIFTS) analysis of 1,8-dihydroxynaphthalene (1,8-DHN) adsorbed on forsterite during UV irradiation at 80 K (**A**) and during heating (from 80 to 300 K) after UV irradiation (**B**). Inset: magnification of the peak at 2340 cm^−1^ due to CO_2_ stretching.

Recently the oxidative polymerization of 1-naphthol (1-HN), 1,8-dihydroxynaphthalene (1,8-DHN), and 1,6-dihydroxynaphthalene (1,6-DHN) adsorbed on forsterite and anatase was investigated following irradiation with UV light (Figure 5) [63]. 

The results of spectral analysis by diffuse reflectance infrared Fourier transform spectroscopy (DRIFTS) coupled with DFT calculations indicate that the oxidative reactivity of hydroxylated naphthalenes is significantly affected by the number and relative disposition of hydroxyl groups. All compounds showed extensive loss of the main vibrational bands, accompanied in the case of DHNs by the formation of new molecular species. Irradiation of 1,8-DHN at 80 K resulted in IR-detectable generation of CO_2_ (2340 cm^−1^), a process previously reported by other authors following irradiation of PAHs in water–ice analogues at 14 K. Notably, little or no reaction was observed under the same conditions on pure powders, without pre-adsorption on minerals. Autoxidation of the compounds adsorbed on forsterite by exposure to ammonia vapors led to band loss and/or broadening due to oxidative polymerization. It was concluded that mineral-surface-promoted oxyPAH photoprocessing is a valuable means of preparing new hybrid materials with properties different from those obtained though usual solution state polymerization chemistry, and which can be investigated for various possible technological applications.

### 3.2. Proton Beam Bombardment on Minerals

Non-terrestrial minerals, such as cosmic dust analogues (CDAs) and meteorites, catalyze the oligomerization of NH_2_COH to a large panel of biologically relevant molecules [64]. CDAs are characterized by amorphous structure, resembling the elemental composition of minerals diffused on the Earth, olivine (MgFeSiO_4_), fayalite (Fe_2_SiO_4_), and forsterite (Mg_2_SiO_4_). CDAs efficiently absorb NH_2_COH to yield pyrimidine nucleobases at 110 °C, simultaneously decreasing the oligonucleotide instability toward the hydrolysis of the phosphodiester bond. In this latter case, the presence of iron (as in the case of olivine and fayalite) was essential for the formation of uracil and cytosine, while forsterite, which contains only magnesium, was not reactive. CDAs were more reactive and selective than their terrestrial counterparts [65].

Meteorites further increased the panel of biomolecules obtainable from NH_2_COH [66]. Purine and pyrimidine nucleobases, including the components of RNA and DNA molecules (adenine, guanine, uracil, thymine, and cytosine), amino acids, carboxylic acid intermediates of ancient cellular metabolic cycles (e.g., Krebs cycle), and reactive condensing agents (e.g., carbodiimide) have been simultaneously synthesized from NH_2_COH at 110 °C in the presence of different types of meteorites [67]. The reaction was also effective in aqueous medium, mimicking primitive ocean or volcanic thermal lake scenarios [68]. The best results were further obtained by high energy proton beam irradiation of NH_2_COH at low temperatures in the presence of meteorites [69]. Within the solar system, high energy proton beams are generated by the Sun as the consequence of flares and coronal mass ejections (solar wind) [70]. In the presence of meteorites (iron, stony-iron, chondrite, and achondrite types) and high-energy protons (170 MeV, linear energy transfer LET 0.57 keV/µm, 243 K), NH_2_COH afforded unprecedented panels of biomolecules, including, most notably, three ribonucleosides, uridine, cytidine, adenosine, and one 2′-deoxyribonucleoside, thymidine. The reaction mechanisms for these transformations have been studied by both computational and analytical tools [71,72,73], encompassing the in situ formation of formaldehyde, formic acid, cyanide, and ammonia, acting as reactive intermediates for the synthesis of sugars, nucleobases (including nucleosides), carboxylic acids, and amino acids, respectively. 

**Figure 6:**
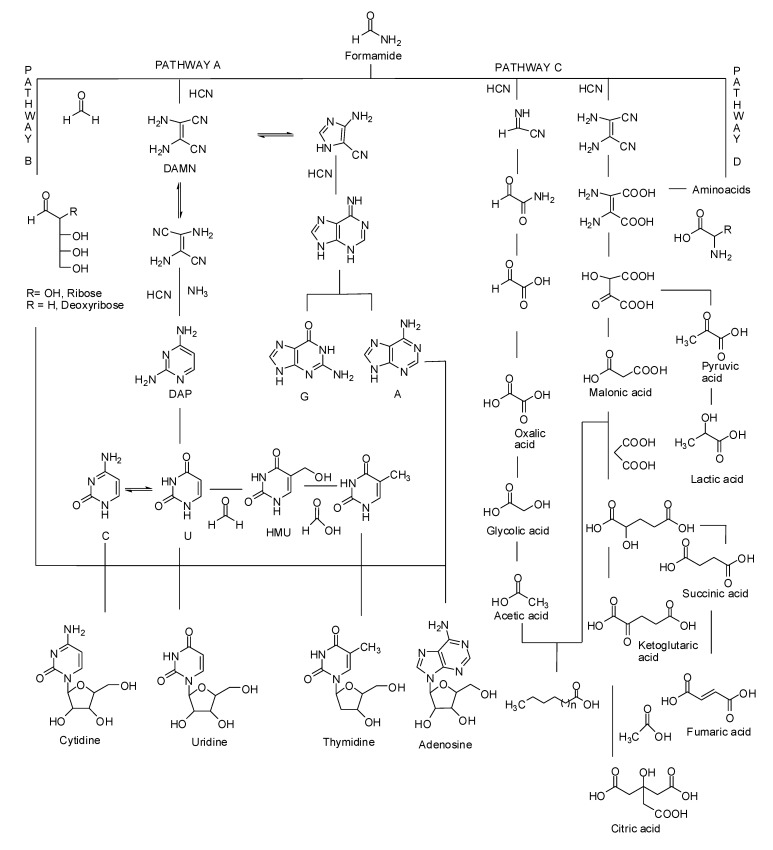
Cascade of chemical transformations affording biomolecules from formamide under proton irradiation conditions in the presence of meteorites. (Pathway A) HCN-mediated formation of nucleobases and nucleotides. (Pathway B) Formaldehyde-mediated aldolformose-like condensation to sugars. (Pathway C) HCN-mediated lactate-like and malonate-like condensations to carboxylic acids and di-carboxylic acids. (Pathway D) Strecker condensation to afford aminoacids.

The different cascades of chemical transformations for the synthesis of products, as suggested on the basis of the detected intermediates, are reported in Figure 6. They include: (a) the formation of nucleobases by initial condensation of formamide and cyanide to yield diaminomalononitrile (DAMN), followed by DAMN transformation to pyrimidine and purine derivatives by two connected pathways involving ammonia, formaldehyde, and formic acid (pathway A) [74]; (b) the formation of sugars and nucleosides by a combined formaldehyde aldol-like condensation and radical coupling (pathway B) [75]; (c) the formation of carboxylic acids by lactate and malonate Claisen-like condensation (pathway C) [76]; and (d) the formation of amino acids by Strecker-like condensation [77]. Meteorites control the regioselectivity and stereochemistry of the products, adsorbing key intermediates and tuning the addition of reagents on the basis of their steric hindrance. For example, the natural β-stereochemistry of nucleotides prevailed during the meteorite-mediated radical coupling of nucleobase onto the sugar moiety, due to the coordination of the geminal hydroxyl moieties of sugar with the metal atoms present on the surface of the mineral. In a similar way, the interaction between the sugar and the mineral surface increased the ribofuranose form with respect to the possible, but useless, pyranose isomer [78]. High energy heavy atoms (e.g., accelerated ^11^B atoms), mimicking cosmic ray radiation, performed in a similar way, extending plausible prebiotic scenarios well beyond our solar system [79].

## 4. Miscellanea

A brief list of systems and processes that have not yet been explored for their actual potential in materials science, but that may be worth pursuing in the quest for innovative properties and technological solutions, is reported herein. 

The seminal Urey and Miller experiment in 1953 paved the way fora number of studies aiming to simulate the conditions of the early Earth (e.g., synthesis and discharge experiments performed in N_2_/CH_4_ mixtures) as well as the atmospheres of other planets and satellites.

Of special interest, in this context, is the atmosphere of Titan, the largest moon of Saturn, which consists of about 98% nitrogen and 2% methane, along with small amounts of H_2_, HCN, CO, and organics such as ethane, ethylene, acetylene, and cyanoacetylene [79]. By solar UV irradiation and energetic particle bombardment, an unusual class of heteropolymer molecules can be produced, which are commonly referred to as tholins [80]. Various approaches have been used to reproduce tholin formation on Titan, including, chiefly cold plasma discharge, hot plasma discharge, UV irradiation, γ-radiation/soft X-rays, and proton and electron bombardment. The resulting tholins display absorption and emission properties that resemble those of Titan’s haze. For example, tholins produced in a DC spark discharge at room temperature exhibited fluorescence under514 nm laser excitation, whereas tholins produced via electric discharge (60 Hz AC) at 195 K produced broad, featureless fluorescence around 471 nm under 410 nm excitation. Chromatographic analysis revealed polar fluorescent species that resulted from exposure to water and/or heat. Most tholins exhibit solubility in polar solvents, whereas tholins produced in a photochemical flow reactor are insoluble in most solvents, indicating that products produced by different techniques differ in their physical and chemical properties.

Current evidence suggest that tholins consist of highly unsymmetrical polycyclic aromatic nitrogenated hydrocarbons (PANHs), conjugated imines, and nitriles with some degree of aromaticity. In particular, they may contain polymers or oligomers of HCN and HC_3_N polymers. Linear and cyclic aminonitriles have been proposed to account for some families of compounds. Average molecular weights are of a few kilodaltons, with peak distribution in mass spectra organized in regular clusters separated by 13 or 14 mass/charge units, suggesting polymeric but not linear structures. Tholins produced by UV irradiation are largely insoluble due to a high degree of cross-linking, while those generated in spark discharges appeared to produce aliphatic mixtures and polyacetylene. Overall, tholin composition may vary significantly depending on:

(i) the methane/nitrogen ratio;
(ii) the energy source;
(iii) the pressure and temperature of the gas mixture;
(iv) oxygen, CO_2_, and water contamination.

Insoluble organic matter (IOM) is a term commonly used to indicate a major constituent of organic matter in meteorites, particularly in carbonaceous chondrites, which is insoluble in water, acids, or organic solvents. In the Murchison meteorite, IOM accounted for more than 90% of the organic carbon, with low amounts of hydrogen, oxygen, nitrogen, and sulfur. IOM seems to consist of aromatic hydrocarbon cores with short aliphatic chains. However, there are grounds to believe that progress in the elucidation and mimicking of IOM production on meteorites, as well as of tholin generation in Titan’s haze, may disclose a hitherto unexplored repertoire of highly tunable organic systems amenable to manipulation and tailoring for various applications, e.g., in surface functionalization, nanoparticle deposition, and UV screening.

Another promising scenario of organic systems and scaffolds is expected to derive from the emerging framework of prebiotic reactions underpinning nucleic acid formation. Herein, only brief mention is given to reactions of cyanamide and glyoxal which proceed through different pathways depending on molar ratios and reaction conditions to afford oligomeric compounds. Condensations conducted in acetone and in aqueous solution have indicated the formation of some repetitive motifs, including a range of heterocyclic scaffolds. Attention has been called to generation of an insoluble black solid from the cyanamide glyoxal (2:1) mixture in aqueous medium. Soluble oligomers were reported to participate in dynamic equilibria, producing thermodynamically stable substances with relatively limited product distributions, suggesting a favorable condition for synthetic efforts toward novel functional materials and systems. Finally, entirely unexplored opportunities may derive from systematic investigations of the chemical and biochemical transformations that may operate in the hydrothermal environment and in the fumaroles of the Phlegrean Fields [81]. The unique set of conditions put together in a volcanic hydrothermal environment is likely to disclose novel types of chemistries and biochemical transformations, with unprecedented characteristics for application in materials science, especially in biomedical and technological settings.

## 5. Conclusions

Materials science is an ever-expanding area of research that has advanced dramatically over the past two decades. As in most other fields of research, further progress is likely to occur only when a truly interdisciplinary approach is implemented. Herein, tackling the secrets of astrochemical and prebiotic processes and the origin of life present an outstanding opportunity. This approach requires not only close interactions between experts from different disciplines, like chemistry, physics, biology, and engineering, but also the optimal integration of experimental and theoretical approaches [82,83,84,85]. The latter are optimally suited to deal with the emergent systems of increasing complexity that characterize prebiotic contexts and that have opened the doorway to systems chemistry [86]. Cutting-edge research on theoretical chemistry benefits from the fast pace of methodological advancements that expand the current scope of computational approaches. These are expected to play a key role in interpreting spectroscopic parameters that are crucial for molecular identification in space and for predicting reaction pathways based on accurate kinetic and thermodynamic analysis of key steps. 

## References

[1] Leger A., Puget J.L. (1984). Identification of the ‘unidentified’ IR emission features of interstellar dust?. Astron. Astrophys..

[2] Allamandola L.J., Tielens A.G.G.M., Barker J.R. (1985). Polycyclic Aromatic Hydrocarbons and the unidentified infrared emission bands: Auto exhaust along the milky way!. Astrophys. J..

[3] Blasberger A., Behar E., Perets H.B., Brosch N., Tielens A.G.G.M. (2017). Observational Evidence Linking Interstellar UV Absorption to PAH Molecules. Astrophys. J..

[4] López-Puertas M., Dinelli B.M., Adriani A., Funke B., García-Comas M., Moriconi M.L., D’Aversa E., Boersma C., Allamandola L.J. (2013). Large abundances of polycyclic aromatic hydrocarbons in Titan’s upper atmosphere. Astrophys. J..

[5] Peeters E., Hony S., Van Kerckhoven C., Tielens A.G.G.M., Allamandola L.J., Hudgins D.M., Bauschlicher C.W. (2002). The rich 6 to 9 µm spectrum of interstellar PAHs. Astron. Astrophys..

[6] Bauschlicher C.W., Peeters E., Allamandola L.J. (2009). The Infrared Spectra of Very Large Irregular Polycyclic Aromatic Hydrocarbons (PAHs): Observational Probes of Astronomical PAH Geometry, Size, and Charge. Astrophys. J..

[7] Rapacioli M., Joblin C., Boissel P., Calvo F., Spiegelman F. (2005). Spectroscopy of Polycyclic Aromatic Hydrocarbons and Very Small Grains in Photodissociation Regions. Astron. Astrophys..

[8] Simon A., Joblin C. (2009). Photodissociation of [Fe_x_(C_24_H_12_)_y_]^+^ Complexes in the PIRENEA Setup: Iron-Polycyclic Aromatic Hydrocarbon Clusters as Candidates for Very Small Interstellar Grains. J. Phys. Chem..

[9] Rosenberg M.J.F., Kazandjian M.V., van der Werf P.P., Israel F.P., Meijerink R., Weiß A., Requena-Torres M.A., Güsten R. (2014). Radiative and mechanical feedback into the molecular gas of NGC 253. Astron. Astrophys..

[10] Galué H.A., DíazLeines G. (2017). Origin of Spectral Band Patterns in the Cosmic Unidentified Infrared Emission. Phys. Rev. Lett..

[11] Parker D.S.N., Zhang F., Kim Y.S., Kaiser R.I., Landera A., Kislov V.V., Mebel A.M., Tielens A.G.G.M. (2012). Low temperature formation of naphthalene and its role in the synthesis of PAHs (Polycyclic Aromatic Hydrocarbons) in the interstellar medium. Proc. Natl. Acad. Sci. USA.

[12] Merino P., Švec M., Martinez J.I., Jelinek P., Lacovig P., Dalmiglio M., Lizzit S., Soukiassian P., Cernicharo J., Martin-Gago J.A. (2014). Graphene etching on SiC grains as a path to interstellar polycyclic aromatic hydrocarbons formation. Nature Commun..

[13] Bouwman J., Mattioda A.L., Linnartz H., Allamandola L.J. (2011). Photochemistry of Polycyclic Aromatic Hydrocarbons in Cosmic Water Ice I. Mid-IR Spectroscopy and Photoproducts. Astron. Astrophys..

[14] Bernstein M.P., Sandford S.A., Allamandola L.J., Gillette J.S., Clemett S.J., Zare R.N. (1999). UV Irradiation of Polycyclic Aromatic Hydrocarbons in Ices: Poduction of Alcohols, Quinones, and Ethers. Science.

[15] Bernstein M.P., Dworkin J.P., Sandford S.A., Allamandola L.J. (2001). Ultraviolet Irradiation of Naphthalene in H2O Ice: Implications for Meteorites and Biogenesis. Meteorit. Planet. Sci..

[16] Cecchini M.M., Reale S., Manini P., d’Ischia M., De Angelis F. (2017). Modeling Fungal Melanin Buildup: Biomimetic Polymerization of 1,8-Dihydroxynaphthalene Mapped by Mass Spectrometry. Chem. Eur. J..

[17] Manini P., Bietti M., Galeotti M., Salamone M., Lanzalunga O., Cecchini M.M., Reale S., Crescenzi O., Napolitano A., De Angelis F. (2018). Characterization and Fate of Hydrogen-Bonded Free-Radical Intermediates and Their Coupling Products from the Hydrogen Atom Transfer Agent 1,8-Naphthalenediol. ACS Omega.

[18] Manini P., Lino V., Franchi P., Gentile G., Sibillano T., Giannini C., Picardi E., Napolitano A., Valgimigli L., Chiappe C. (2019). A Robust Fungal Allomelanin Mimic: An Antioxidant and Potent π-Electron Donor with Free-Radical Properties that can be Tuned by Ionic Liquids. ChemPlusChem.

[19] Longo D.L., Stefania R., Aime S., Oraevsky A. (2017). Melanin-Based Contrast Agents for Biomedical Optoacoustic Imaging and Theranostic Applications. Int. J. Mol. Sci..

[20] Pezzella A., Barra M., Musto A., Navarra A., Alfè M., Manini P., Parisi S., Cassinese A., Criscuolo V., d’Ischia M. (2015). Stem cell-compatible eumelaninbiointerface fabricated by chemically controlled solid state polymerization. Mater. Horiz..

[21] Ferris J.P., Hagan W.J. (1984). HCN and chemical evolution: The possible role of cyano compounds in prebiotic synthesis. Tetrahedron.

[22] Matthews C.N., Minard R.D. (2006). Hydrogen cyanide polymers, comets and the origin of life. Faraday Discuss..

[23] Miller S.L. (1953). A production of amino acids under possible primitive earth conditions. Science.

[24] Sanchez R.A., Ferris J.P., Orgel L.E. (1967). Studies in prebiotic chemistry. II Synthesis of purine precursors and amino acids from aqueous hydrogen cyanide. J. Mol. Biol..

[25] Raulin F., Fonsalas F., Wolny M. (1984). Aminomalononitrile: Some new data of prebiotic interest. Orig. Life.

[26] Thissen H., Koegler A., Salwiczek M., Easton C.D., Qu Y., Lithgow T., Evans R.A. (2015). Prebiotic-chemistry inspired polymer coatings for biomedical and materials science applications. NPG Asia Mater..

[27] Menzies D.J., Ang A., Thissen H., Evans R.A. (2017). Adhesive prebiotic chemistry inspired coatings for bone contacting applications. ACS Biomater. Sci. Eng..

[28] Lee H., Dellatore S.M., Miller W.M., Messersmith P.B. (2007). Mussel-inspired surface chemistry for multifunctional coatings. Science.

[29] Kang S.M., Rho J., Choi I.S., Messersmith P.B., Lee H. (2009). Norepinephrine: Material independant, multifunctional surface modification reagent. J. Am. Chem. Soc..

[30] d’Ischia M., Palumbo A., Prota G. (1988). Adrenalinoxidationrevisited. New products beyond the adrenochrome stage. Tetrahedron.

[31] Ball V., Toh R.J., Voelcker N., Thissen H., Evans R. (2008). Electrochemical deposition of aminomalonotrile based films. Colloids Surf. A.

[32] Saitta A.M., Saija F. (2014). Miller experiments in atomistic computer simulations. Proc. Natl. Acad. Sci. USA.

[33] Adande G.R., Woolf N.J., Ziurys L.M. (2013). Observations of interstellar formamide: Availability of a prebiotic precursor in the galactic habitable zone. Astrobiology.

[34] Saladino R., Crestini C., Pino S., Costanzo G., Di Mauro E. (2012). Formamide and the origin of life. Phys. Life Rev..

[35] Saladino R., Botta G., Pino S., Costanzo G., Di Mauro E. (2012). Genetics first or metabolismfirst? The formamide clue. Chem. Soc. Rev..

[36] Šponer J.E., Šponer J., Nováková O., Brabec V., Šedo O., Zdráhal Z., Costanzo G., Pino S., Saladino R., Di Mauro E. (2016). Emergence of the First Catalytic Oligonucleotides in a Formamide-Based Origin Scenario. A review. Chem. Eur. J..

[37] Saladino R., Botta G., Bizzarri B.M., Di Mauro E., Garcia Ruiz J.M. (2016). A global scale scenario for prebiotic chemistry: Silica-based self-asssembled mineral structures and formamide. Biochemistry.

[38] Gull M. (2014). Prebiotic Phosphorylation Reactions on the Early Earth. Challenges.

[39] Yamagata Y., Matsukawa T., Mohri T., Inomata K. (1979). Phosphorylation of adenosine in aqueous solution by electric discharges. Nature.

[40] Schwartz A., Ponnamperuma C. (1968). Phosphorylation on the Primitive Earth: Phosphorylation of Adenosine with Linear Polyphosphate Salts in Aqueous Solution. Nature.

[41] Graaf R., Visscher J., Schwartz A.W. (1995). A plausibly prebiotic synthesis of phosphonic acids. Nature.

[42] Benner S.A., Kim H.J., Carrigan M.A. (2012). Asphalt, Water, and the Prebiotic Synthesis of Ribose, Ribonucleosides, and RNA. Acc. Chem. Res..

[43] Schoffstall A.M., Mahone S.M. (1988). Formate ester formation in amide solutions. Orig. Life Evol. Biosph..

[44] Schoffstall A.M., Laing E.M. (1985). Phosphorylation mechanisms in chemical evolution. Orig. Life Evol. Biosph..

[45] Yamagata Y., Watanabe H., Saitoh M., Namba T. (1991). Volcanic production of polyphosphates and its relevance to prebiotic evolution. Nature.

[46] Costanzo G., Saladino R., Crestini C., Ciciriello F., Di Mauro E. (2007). Nucleoside Phosphorylation by PhosphateMinerals. J. Biol. Chem..

[47] Mamajanov I., Engelhart A.E., Bean H.D., Hud N.V. (2010). DNA and RNA in anhydrous media: Duplex, triplex, and G-quadruplex secondary structures in a deep eutectic solvent. Angew. Chem. Int. Ed..

[48] Ciaramella M., Cannio R., Moracci M., Pisani F.M., Rossi M. (1995). Molecular biology of extremophiles. World J. Microbiol. Biotechnol..

[49] Rimmer P.B., Shorttle O. (2019). Origin of Life’s Building Blocks in Carbon- and Nitrogen-Rich Surface Hydrothermal Vents. Life.

[50] van de Vossenberg J.L., Driessen A.J., Konings W.N. (1998). The essence of being extremophilic: The role of the unique archaeal membrane lipids. Extremophiles.

[51] de Rosa M., Gambacorta A., Millonig G., Bu’Lock J.D. (1974). Convergent characters of extremely thermophilic acidophilic bacteria. Experientia.

[52] Guagliardi A., Cerchia L., Moracci M., Rossi M. (2000). The chromosomal protein sso7d of the CrenarchaeonSulfolobus solfataricus rescues aggregated proteins in an ATP hydrolysis-dependent manner. J. Biol. Chem..

[53] Pouwels J., Moracci M., Cobucci-Ponzano B., Perugino G., Van der Oost J., Kaper T., Lebbink J., de Vos W., Ciaramella M., Rossi M. (2000). Activity and stability of hyperthermophilic enzymes: A comparative study on two archaeal ß-glycosidases. Extremophiles.

[54] Aguilar C., Sanderson I., Moracci M., Ciaramella M., Nucci R., Rossi M., Pearl L.H. (1997). Crystal structure of the ß-glycosidase from the hyperthermophilic Archaeon Sulfolobussolfataricus: Resilience as a key factor in thermostability. J. Mol. Biol..

[55] Atalah J., Cáceres-Moreno P., Espina G., Blamey J.M. (2019). Thermophiles and the applications of their enzymes as new biocatalysts. Bioresour. Technol..

[56] Ariel S., Askari S., Evans S.V., Hwang C., Jay J., Scheffer J.R., Trotter J., Walsh L., Wong Y.F. (1987). Reaction selectivity in solid state photochemistry. Tetrahedron.

[57] Lange R.Z., Hofer G., Weber T., Schlüter A.D. (2017). A Two-Dimensional Polymer Synthesized through Topochemical [2 + 2]-Cycloaddition on the MultigramScale. J. Am. Chem. Soc..

[58] Sonoda Y. (2011). Solid-State [2 + 2] Photodimerization and Photopolymerization of α,ω-Diarylpolyene Monomers: Effective Utilization of Noncovalent Intermolecular Interactions in Crystals. Molecules.

[59] Barentsen H.M., van Dijk M., Zuilhof H., Sudhölter E.J.R. (2000). Thermal and Photoinduced Polymerization of Thin Diacetylene Films. 1. Phthalimido-Substituted Diacetylenes. Macromolecules.

[60] Badarau C., Wang Z.Y. (2004). Synthesis and Optical Properties of Thermally and Photochemically Cross-Linkable Diacetylene-Containing Polymers. Macromolecules.

[61] Matsumoto A., Odani T. (2001). Topochemical Polymerization of 1,3-Diene Monomers and Features of Polymer Crystals as Organic Intercalation Materials. Macromol. Rapid Commun..

[62] Matsumoto A. (2001). Stereospecific Polymerization of 1,3-Diene Monomers in the Crystalline State. Progr. React. Kinet. Mech..

[63] Potenti S., Manini P., Fornaro T., Poggiali G., Crescenzi O., Napolitano A., Brucato J.R., Barone V., d’Ischia M. (2018). Solid State Photochemistry of HydroxylatedNaphthalenes on Forsterite: Probing Polycyclic Aromatic Hydrocarbon-Mineral Interactions under Astrochemically-Relevant UV Irradiation Conditions. ACS Earth Space Chem..

[64] Carota E., Botta G., Rotelli L., Di Mauro E., Saladino R. (2015). Current Advances in Prebiotic Chemistry Under Space Conditions. Curr. Org. Chem..

[65] Saladino R., Crestini C., Neri V., Brucato J.R., Colangeli L., Ciciriello F., Di Mauro E., Costanzo G. (2005). Synthesis and Degradation of Nucleic Acid components by formamide and cosmic dust analogues. ChemBioChem.

[66] Saladino R., Crestini C., Cossetti C., Di Mauro E., Deamer D. (2011). Catalytic effects of Murchison Material: Prebiotic Synthesis and Degradation of RNA Precursors. Orig. Life Evol. Biosph..

[67] Saladino R., Botta G., Delfino M., Di Mauro E. (2013). Meteorites as catalysts for prebiotic chemistry. Chem. Eur. J..

[68] Rotelli L., Trigo-Rodríguez J.M., Moyano-Cambero C.M., Carota E., Botta L., Di Mauro E., Saladino R. (2016). The key role of meteorites in the formation of relevant prebiotic molecules in a formamide/water environment. Sci. Rep..

[69] Saladino R., Carota E., Botta G., Kapralov M., Timoshenko G.N., Rozanov A.Y., Krasavin E., Di Mauro E. (2015). Meteorite-catalyzed syntheses of nucleosides and of other prebiotic compounds from formamide under proton irradiation. Proc. Natl. Acad. Sci. USA.

[70] Dalgarno A. (2012). The galactic cosmic ray ionization rate. Proc. Natl. Acad. Sci. USA.

[71] Hudson J.S., Eberle J.F., Vachani R.H., Rogers L.C., Wade J.H., Krishnamurthy R., Springsteen G.A. (2012). A unified mechanism for abiotic adenine and purine synthesis in formamide. Angew. Chem. Int. Ed..

[72] Jeilani Y.A., Nguyen H.T., Newallo D., Dimandja J.M.D., Nguyen M.T. (2013). Free radical routes for prebiotic formation of DNA nucleobases from formamide. Phys. Chem. Chem. Phys..

[73] Ferus M., Michalcikovà R., Shestivskà V., Sponer J., Sponer J.E. (2014). High Energy chemistry of formamide: A simpler way for nucleobase formation. J. Phys. Chem. A.

[74] Ferus M., Civis S., Mladek A., Sponer J., Juha L., Sponer J.E. (2012). On the road from formamide ices to nucleobases: IR spectroscopic observation of a direct reaction between cyano radicals and formamide in a high-energy impact event. J. Am. Chem. Soc..

[75] Saladino R., Bizzarri B.M., Botta L., Šponer J., Šponer J.E., Georgelin T., Jaber M., Rigaud B., Kapralov M., Timoshenko G.N. (2017). Proton irradiation: A key to the challenge of N-glycosidic bond formation in a prebiotic context. Sci. Rep..

[76] Botta L., Bizzarri B.M., Piccinino D., Fornaro T., Brucato J.R., Saladino R. (2017). Prebiotic synthesis of carboxylic acids, amino acids and nucleic acid bases from formamide under photochemical condition. Eur. Phys. J. Plus.

[77] Saladino R., Botta L., Di Mauro E. (2018). The Prevailing Catalytic Role of Meteorites in Formamide Prebiotic Processes. Life.

[78] Saladino R., Carota E., Botta G., Kapralov M., Timoshenko G.N., Rozanov A., Krasavin E., Di Mauro E. (2016). First evidence on the role of heavy ions irradiation of meteorites and formamide in the origin of biomolecule. Orig. Life Evol. Biosph..

[79] Niemann H.B., Atreya S.K., Bauer S.J., Carignan G.R., Demick J.E., Frost R.L., Gautier D., Haberman J.A., Harpold D.N., Hunten D.M. (2005). The abundances of constituents of Titan’s atmosphere from the GCMS instrument on the Huygens probe. Nature.

[80] Waite Jr J.H., Young D.T., Cravens T.E., Coates A.J., Crary F.J., Magee B., Westlake J. (2007). The Process of Tholin Formation in Titan’s Upper Atmosphere. Science.

[81] Botta L., Saladino R., Bizzarri B.M., Cobucci-Ponzano B., Iacono R., Avino R., Caliro S., Carandente A., Lorenzini F., Tortora A. (2018). Formamide-basedprebioticchemistry in the PhlegreanFields. Adv. Space Res..

[82] Puzzarini C., Barone V. (2018). Diving for Accurate Structures in the Ocean of Molecular Systems with the Help of Spectroscopy and Quantum Chemistry. Acc. Chem. Res..

[83] Puzzarini C., Barone V. (2019). A never-ending story in the sky: The secrets of chemical evolution. Phys. Life Rev..

[84] Vallance C. (2017). Astrochemistry: From the Big Bang to the Present Day.

[85] Islam S., Powner M.W. (2017). Prebiotic system chemistry: Complexity overcoming clutter. Chem.

[86] Ashkenasy G., Hermans T.H., Ottoc S., Taylor A.F. (2017). Systems Chemistry. Chem. Soc. Rev..

